# Heavy metal toxicity arising from the industrial effluents repercussions on oxidative stress, liver enzymes and antioxidant activity in brain homogenates of *Oreochromis niloticus*

**DOI:** 10.1038/s41598-023-47366-4

**Published:** 2023-11-15

**Authors:** Sarwat Ishaq, Ghazala Jabeen, Mateen Arshad, Zakia Kanwal, Fakhar Un Nisa, Rida Zahra, Zunaira Shafiq, Hassan Ali, Khush Bakht Samreen, Farkhanda Manzoor

**Affiliations:** 1 Department of Zoology, Lahore College for Women University, Lahore, Pakistan; 2 Punjab Wildlife and Parks Department, Lahore, Pakistan

**Keywords:** Animal behaviour, Animal physiology, Diagnostic markers

## Abstract

Industrial effluents reaching to the aquatic ecosystem is one of the major causes of environmental pollution and exposure to industrial effluents containing harmful substances may be a serious threat to human health. Therefore, the present study aimed to determine the sub-lethal (1/5th of predetermined LC_50_) impact of industrial effluents from Sundar Industrial Estate on *Oreochromis niloticus* with proper negative control. The physicochemical analysis of industrial effluents showed enormous loads of inorganic pollutants and exhibited high mean levels of heavy metals, Mn, Fe, Pb, Ni, Cr, Hg, As, Zn and Fe with statistically significant differences at *p* < 0.05. Highest level of Mn and Fe was detected in effluent’s samples as 147.36 ± 80.91 mg/L and 90.52 ± 32.08 mg/L, respectively. Exposure led to increase in serum biochemical parameters alanine aminotransferase + 25%, aspartate aminotransferase + 20% and alkaline phosphatase + 7% over control although superoxide dismutase, catalase, glutathione peroxidase and reduced glutathione significantly increased as 3.42, 2.44, 4.8 and 8 folds, respectively in metabolically active tissue brain which indicated stress caused by industrial effluents. The results concluded that industrial effluent has potent oxidative stress inducers on one hand whereas histoarchitectural and physiological toxicity causing contaminants on the other. This condition may adversely affect the health of aquatic organisms, the fish and ultimately the human beings.

## Introduction

Industrial wastes contribute colossal quantities of organic and inorganic contaminants whereas the treatment processes of industrial discharges have not been practiced due to the intensification and growth of industries. The toxic chemicals present in these waste has severe toxic potential to affect the aquatic life and pose a serious threat to human health directly or indirectly, thereby, disrupting the whole systems and environment^1,2^. Generally, the large number of industries in the developing countries dump their wastes without proper treatment at a large scale that comprise of massive quantities of heavy metals, oil/ grease, dissolved/suspended solids, high sulphates and nitrates loads organic chemicals, toxic phenolic compounds that cause considerable organic and inorganic pollution with significant deleterious effects on the fish and aquatic biota residing these waste contaminated water bodies^3^.

Heavy metals like zinc, copper, cobalt, iron and manganese etc. are required by the aquatic organisms in trace amounts but elevated levels beyond the permissible limits are toxic while other elements have deleterious effects and exhibit adverse changes in negligible amount. These potentially harmful and toxic elements released into the aquatic environment negatively impact the organisms and aquatic ecosystems due to their non-biodegradable nature, persistence, toxicity, oxidative stress, bio-accumulative properties and biomagnification along the food chains when eventually assimilated by the humans’ cause subsequent health risks and detrimental effects. The concern on detrimental effects of industrial effluents is growing due to the lack of proper disposable strategies, poor control, inadequate or partial treatment procedures specifically in the developing and underdeveloped countries^4^.

Liquid effluents and raw industrial wastewater is generated continuously in the massive quantities during industrial activities with a varied composition. So, industrial effluents have diverse composition both in terms of quality and quantity of pollution load^5^. Aquatic plants, submerged vegetation, benthic organisms and sediments accumulate the pollutants that ultimately enter the aquatic environment through natural, industrial and anthropogenic activities in fish tissues and higher trophic levels via the aquatic food chain^6^, therefore, water, aquatic biota and fishes have been frequently used as indicator of water pollution and have been proved as the biomarker of pollution and monitoring studies^7^.

A bulk of inorganic toxicants and detrimental chemicals released in the freshwater bodies cause lethal changes through bioaccumulation, biomagnification, cytotoxicity and histopathological alteration in the structure of vital body organs as indicative of oxidative stress and even sudden mortality of the aquatic organisms^8^. The evaluation of sublethal toxic effects on biomarker organisms is the most useful tool for the assessment of ecological health. The fish is the most vulnerable aquatic organisms as major recipient of these toxic chemicals. Several vital body organs, essential tissues, antioxidant enzymes, physiological functions, resistance to pathogens and infections are heavily impacted by the sublethal toxicity. Hence, numerous biomarker approaches and significant changes in the biochemical profile of fish are frequently employed for the evaluation of adverse toxic effects of pollutants^9^.

Liver is the vital body organ of organisms as the controlling and regulating center of all the metabolic activities. The exposure of liver to all the toxicants entering in the organisms, blood flow and its central role in the detoxification and elimination of toxic substances makes it more susceptible to injuries and damages through exposure to the chemicals from the environment. The changes in the liver enzymes, Aspartate amino transferase (AST) and Alanine amino transaminase (ALT) that catalyze the transamination reactions can be used as toxicity biomarkers to elucidate examine cell injury, physiological impairment of tissues and hepatotoxicity. Moreover, the determination of alkaline phosphatase (ALP) provide holistic approach for tissue impairments, cellular damage and histopathological anomalies that can be interconnected to visualize the wide-ranging damage and toxicity^10^. The analysis of biochemical composition of fish is also indicator of probable damage and adverse toxicity. The alteration in levels of various enzymes are considered as early warning signs prior to the hazardous effects occur in fish and biochemical indicators for the testing of water pollution^11^.

Oxidative stress is caused by an imbalance in the generation and removal of reactive oxygen species (ROS). Fish frequently face challenges of toxic and harmful pollutants in aquatic environments, leading to environmental oxidative stress. Fish use antioxidant defense systems to scavenge ROS. Elevated ROS impairs mitochondrial function and leads to excessive opening of mitochondrial channels^12^. Excessive O_2_^•−^ efflux from mitochondria endangers the health of fish. In the antioxidant system, SOD converts O2^•−^ to H_2_O_2_, and the resulting H_2_O_2_ can be reduced by CAT and GSH. In order to maintain ROS within physiological limits, an antioxidant system includes enzymatic (SOD, CAT and GPx) and non-enzymatic (GSH) components^13^. There are the first line of the antioxidant defense system against ROS^14^. Fish under stress of toxicants have been observed to have changes in these enzymes in a variety of tissues when exposed to various industrial effluents in the past^15^,^16^ and^13^.

Biomarkers of contamination exposure in species of fish are crucial indicators if we are to maintain a profitable fishery and a healthy product for men consumption and health^17^. Due to their capacity to concentrate, store, and metabolize water-borne toxins, aquatic species have been recognized as an excellent and economical model system for assessing the hazardous potential of pollutants^18^. Therefore, in the current study, we aimed to evaluate the changes in the activity of the most significant antioxidant system (CAT, SOD, GPx, and GSH) in the brain related to oxidative stress as well as biochemical analysis of the liver, hematological, and histopathological in fish, *O. niloticus* exposed to 1/5th predetermined LC_50_ of composite of raw effluent of different industries for monitoring the wastewater toxicity in a holistic manner to evaluate cytotoxicity, hepatotoxicity, physiological disturbances, oxidative stress in fish and subsequently potential damages to humans and public health.

## Materials and methods

### Study area

**Figure 1:**
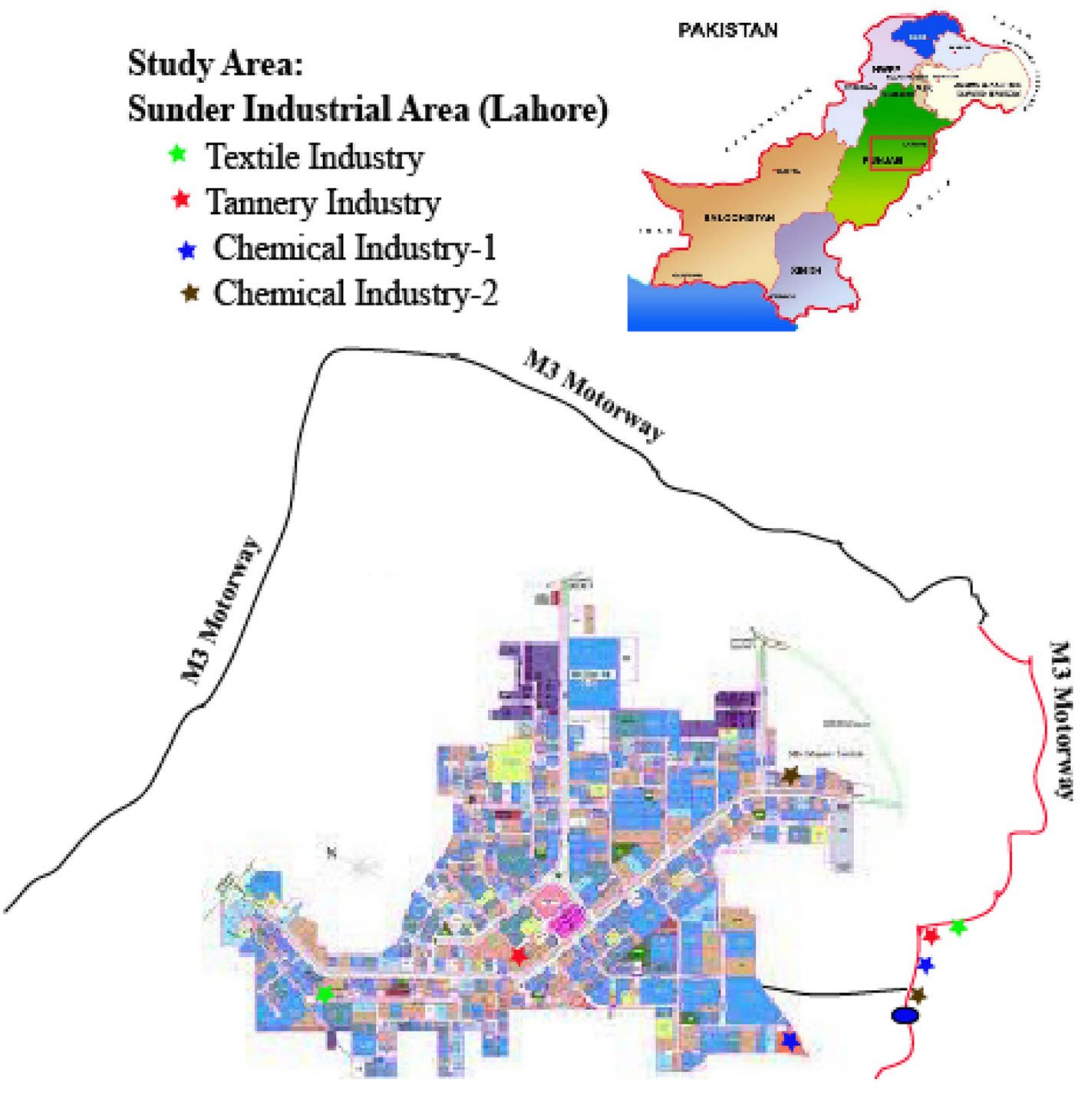
Geographical map of the study area.

The study area selected for the sampling of industrial effluents was Sundar Industrial Estate (SIE), one of Lahore's most rapidly developing and polluted industrial regions. The industrial region covers 1750 acres of land and is home to 400 major and medium sized businesses such as engineering, chemical, paint, pharmaceutical, textile, and food businesses as illustrated in Fig. 1.

The study region is located between 31.2883° N and 74.1739° E. Wastewater from these industries is continually discharged into the adjacent areas on a regular basis. The people who live in the vicinity of these locations are at a risk of being polluted by the environment.

The four sub-sampling sites, consisting of Textile, Tannery and two chemical industries were carefully chosen for the collection of industrial effluents. The samples were collected by using clean pair of new, non-powdered, disposable gloves and 4 sample containers of 5 L capacity were filled directly from each fixed sub-sampling site according to the guidelines of^19^.

### Characterization of industrial effluents

Effluent samples collected from all the sampling sites were analyzed for heavy metals, chlorides, ammonia, sulphates, nitrate and phosphate etc. by using Whole Effluent Toxicity testing (WET)^20^.

### Physico-chemical analysis of the industrial effluents

The physical and chemical analysis of the parameters: potential hydrogen** (**pH), electrical conductivity (EC), total dissolved solid (TDS), total Salinity, carbon dioxide (CO_2_), chlorides, magnesium (Mg), calcium (Ca), total Alkalinity, carbon alkalinity, phenolphthalein alkalinity (P. Alkalinity) and total hardness, by using standard methods for Examination of Water and Wastewater"^21^. The temperature was measured by thermometer whereas the pH, EC and TDS were calculated by digital pH meter (JENWAY 3505 pH meter) and EC meter/TDS meter (JENCO 3173 COND), respectively. Chlorides in the industrial effluent samples were analyzed by mercuric nitrate method.

### Chemical analysis of industrial effluents for heavy metal determination

The Industrial samples were brought in the laboratory and then performed wet Digestion for heavy metal analysis. The digestion was performed by taking 10 ml of sample, 15 ml of conc. HNO_3_ and 5 ml of conc. HCl (1:3). This mixture was swirled gently, covered with watch glass and left at room temperature for about an hour. Samples were then heated on hot plate until yellow fumes were released and the solution became clear. After cooling, the acid solution was filtered by Millipore filter (0.45 μm) and volume was made up to 150 ml by double distilled deionized water^22^. Different concentrations of standard solutions for every metal were prepared. Atomic Absorption Spectrophotometer (AAS, Z-5000, and Polarized Zeeman) was used for heavy metal analysis. All the lamps used were hollow cathode lamps (TJA Solutions, UK) and the air and acetylene mixture was the fuel supply.

### Experimental fish, experimental studies, methods, relevant guidelines and regulations

The animal study protocol and methods were approved by the Research Ethical Review committee, Institutional Review Board, ASRB (Advanced Studies and Research Board) and Ethics Committee (REF No/LCWU/Zoo/546). The AVMA guidelines for the euthanasia of the animals as an approach to follow human endpoints and the ARRIVE guidelines were also followed during the investigation and experimentation. In addition to these guidelines, all the methods were performed in accordance with the recommended relevant international guidelines and regulations.

#### Evaluation of sublethal toxicity of industrial effluents

Healthy juveniles of (n = 120), *Oreochromis niloticus* with an average weight of (10 ± 2 g) and length (3 ± 0.5 inch) were used for the sublethal toxicity trials. Fish were kept in tanks filled with dechlorinated tap water for seven days to allow them to adjust to their new surroundings. Fish were fed a 40% crude protein diet from Oryza Organics Pvt. (Ltd.) at 3% body weight on a daily basis, twice a day (morning at 9:00am and evening at 17:00 pm), with uneaten food being siphoned off on a frequent basis to prevent the accumulation of metabolites. Following acclimatization, fish were divided into two groups; control and treatment group. Control fish was not exposed to industrial effluents whereas the treated fish was exposed to 1/5th of the predetermined LC_50_ concentrations. The effluents collected from different industries e.g. textile industry, tanning industry and chemical industry were mixed in equal quantities to make a composite industrial effluent sample for the sublethal exposure of fish and the required exposure concentrations were prepared by following the methods of^23^. The predetermined LC_50_ concentration of 18.311% was used for the sublethal (1/5th of the LC_50_ concentrations) exposure for a period of 15 days. The sub-lethal exposure trial to Nile tilapia (*Oreochromis niloticus*) for 1/5th of LC_50_ were conducted by following the US EPA,^19^.

### Biochemical, hematological, antioxidant and histological analysis

The fish (n = 10) were sampled per group, per time were humanely sacrificed by following AVMA at the end of the exposure period by following the^24^ guidelines for the euthanasia of fish using clove oil which is a natural product. Blood from the fish was collected using disposable hypodermic syringes and placed in EDTA tubes for hematological analysis. While 1.5 ml blood was taken in clot activators for metabolic enzyme activity. Sera were separated by centrifugation (after allowing the blood samples to clot at ambient temperature) at 4000 g for 15 min at 4 °C and the supernatants were collected, and frozen at − 80 °C which was used for the biochemical analysis. Portions of the brain and liver of the fish were removed immediately after blood collection, washed in chilled PBS and further processed for enzymatic analysis.

Brain of exposed fish along with control were surgically removed and rinsed in 0.69% cold saline, at 4–6 °C and blotted dry. The tissues were weighed and homogenized (10%, w/v) in 50 mM sodium phosphate buffer (pH 8.0) containing 0.1% Triton X-100 using Potter–Elvehjam homogenizer fitted with a Teflon-coated pestle under ice cold condition. For 30 min, the homogenates were kept in cold with intermittent stirring and centrifuged at 4 °C for 30 min at 10,000 ​g in a refrigerated centrifuge (Sigma, Model: 3K30 St. Louis, USA). The corresponding supernatants were used either fresh for determination for antioxidant assays. Antioxidant enzymes (CAT, SOD, GPx and GSH) were determined by following^25^.

The metabolic enzyme activities of alanine aminotransferase (ALT), aspartate aminotransferase (AST) and Alkaline phosphate (ALP) were evaluated using commercially available “Cromatest Kits” by Linear Chemicals, S.L.U. Spain with Reference numbers as 1,105,000, 1,109,000 and 1,102,015, respectively, while other parameters like urea and plasma glucose were determined by commercially available kits from ARENA BioScien and Human diagnostic worldwide with reference numbers as BS.1/UR02.050.0100 and 10,260, respectively. Moreover albumin and total protein was observed by commercially available kits from BioResearch with code numbers CS001 and CS015, respectively. Analysis of all biochemical parameters was done with a biochemical-type automated analyzer (metrolab 1600 DR clinical chemistry analyzer) by following the protocol of the manufacture. Although globulin fraction was calculated by subtracting the albumin from the total protein^26^.

Hematological indices including Red blood cell (RBC) count, hemoglobin (Hb) content, percentage hematocrit (Ht), mean corpuscle hemoglobin concentration (MCHC), mean corpuscle volume (MCV), mean corpuscle hemoglobin (MCH), platelets, total white blood cell (WBC) count and lymphocytes in the treatment and control groups by following the methods of^25^.

The kidneys and hearts of the fish were removed carefully and preserved in Bouin’s fluid overnight before being dehydrated in ascending degrees of alcohol and cleaned in xylene and 4–6 µ sections were stained by the Hematoxylin and Eosin stain^27^. Then images of these sections were captured by a florescent microscope Euromex, Holland.

### Statistical analysis

Statistical software graphpad prism 10 and SPSS version 21.0 were used for analysis of variance (ANOVA) to compare the mean values of all parameters under study at *p* < 0.05 level of significance using Tukey’s HSD as the post hoc test. All data were presented as mean ± SD. To generate the mean and standard deviation for the data sets, descriptive statistics were used.

### Ethical approval

(294). Fish handling, blood sampling and fish sacrifice during the current investigation was approved by the Ethical Research Review Committee (RERC) of Department of Zoology, Lahore College for Women University.

## Results

### Characterization of industrial effluents

The characterization of the industrial effluents by effluent analysis involves the techniques, approaches and methods revealed and validated in the literature since 1975. The effluent analysis and characterization are categorized into following main groups:

- Analysis of physical and chemical properties of effluents
- Inorganic compounds analysis
- Heavy metals analysis

#### Analysis of physical and chemical properties of effluents

Present study was conducted to evaluate the toxic impact of industrial effluents of Sunder Industrial Estate. Physicochemical analysis revealed that these effluents were highly toxic, not only to aquatic organisms but the entire web chain including human beings being the primary consumers of fish. During current investigation, the results of physicochemical parameters of effluents from the textile industry were highly alkaline having high pH, total alkalinity, Ca, Mg and Chlorides contents whereas tannery industry effluents were mainly acidic in nature and exhibited low values of pH. Analysis of physicochemical parameters, temperature, pH, Free CO_2_, alkalinity as CaCO_3_, Total alkalinity, total hardness, chloride, electrical conductivity, total dissolved solids (TDS) and salinity of control and all industries is presented in Tables 1 and 2.

**Table 1:** Analysis of physical parameters of industrial effluents of sunder industrial estate, Lahore.

Physical parameters	Textile industry	Tannery industry	Chemical industry-I	Chemical industry-II	Control
Color	Light brown	Dark brown	Bluish grey	Brown	Clear
Odor	Unpleasant	Foul smell	Foul smell	Unpleasant	No smell

**Table 2:** Analysis of chemical parameters of industrial effluents of sunder industrial estate, Lahore.

Chemical parameters	Textile industry	Tannery industry	Chemical industry-I	Chemical industry-II	Control
Temperature (°C)	29.00 ± 1.00^a^	30.00 ± 1.00^a^	30.00 ± .1.30^a^	28.00 ± 1.00^a^	26.00 ± 5^a^
pH	8.90 ± 0.51^a^	6.80 ± 0.43^c^	7.60 ± 0.30^b^	7.10 ± 0.08^b^	7.00 ± 0.5^b^
Electrical Conductivity (μ S cm^−1^)	1629 ± 20.53^b^	1686.33 ± 13.76^b^	1352 ± 10.66^c^	1961 ± 42.96^a^	627.67 ± 2.52^d^
TDS (mg L^−1^)	1401.13 ± 50.42^a^	1393.5 ± 37.96^a^	919.56 ± 10.08^b^	1686.73 ± 36.64^a^	356.87 ± 303.86^c^
Total Salinity (mg L^−1^)	1.37 ± 0.25^b^	1.38 ± 0.27^b^	6.3 ± 1.20^a^	1.66 ± 0.13^b^	5.33 ± 1^a^
CO_2_ (mg L^−1^)	200 ± 4.47^a^	200 ± 4.47^a^	280 ± 2.25^a^	220 ± 22.36^a^	200 ± 5^a^
Chloride (mg L^−1^)	1250.64 ± 150.93^a^	445.17 ± 57.64^c^	1000 ± 5.67^b^	347.12 ± 96.51^c^	350 ± 5^c^
Mg (mg L^−1^)	346.67 ± 18.71^ab^	90 ± 98.64^c^	250 ± 3.40^bc^	460.83 ± 88.68^a^	220 ± 5^bc^
Ca (mg L^−1^)	370 ± 11.83^b^	720 ± 16.29^a^	380 ± 2.25^b^	333.33 ± 36.97^b^	213.33 ± 12.58^c^
Total Alkalinity (mg L^−1^)	807 ± 21.32^c^	1111 ± 14.21^a^	700 ± 18.50^d^	909.50 ± 28.22^b^	680 ± 5^d^
Carbon Alkalinity (mg L^−1^)	807.50 ± 12.40^b^	204 ± 16.20^d^	559 ± 10.69^d^	909.5 0 ± 28.22^a^	680 ± 5^c^
Total Hardness (mg L^−1^)	716.67 ± 139^ab^	560 ± 44.04^bc^	845 ± 15.57^a^	790 ± 120.58^ab^	420 ± 5.00^c^
Nitrate–N (mg L^−1^)	10.65 ± 0.54^a^	5.31 ± 0.39^ab^	4.67 ± 0.20^ab^	2.59 ± 0.36^c^	10 ± 5^a^
Ammonia (mg L^−1^)	2.5^a^	2.5^a^	2.5^a^	2.5^a^	0.3^b^
Phosphate (mg L^−1^)	2.25 ± 0.15^a^	0.73 ± 0.02^b^	0.62 ± 0.03^bc^	0.53 ± 0.01^c^	0.02 ± 0.01^d^
Sulphates (mg L^−1^)	180 ± 1.37^b^	194 ± 1.69^a^	117 ± 1.00^c^	123 ± 1.50^c^	60 ± 10^d^

Means with different letters in a single row are statistically non-significant at *p* < 0.05.

The mean values of all water quality variables recorded during the present investigation revealed that they all were significantly higher than the standard values. Whereas highest fluctuations were recorded in TDS and alkalinity as CaCO_3_ of control and treated fish. TDS was recorded in tannery industry as 290% that was significantly higher than that of control group. The electrical conductivity percentage varied in different effluents as 160%, 169%, 115% and 212% in textile, tannery, and chemical-I and chemical-II, respectively. Highest values of EC were recorded in tannery industry and lowest in chemical Industry-I, respectively. In the present study, the levels of electrical conductivity, total dissolved solids, total salinity, total alkalinity and chlorides determined for the effluents of textile, tannery and chemical industries during the present research endeavor were high than the recommended limits by EPA, WHO and values of normal tap water (Table 3) rendering it unfit for fish culture and human consumption posing threats to public health.

**Table 3:** Suitable water quality parameters and permissible metal levels in relation to fish culture.

Water quality parameters	EPA	WHO standards	Control (normal tap water)
Electric conductivity (µ s cm^−1^)	100–2000 μScm^−1^	400	627.67 ± 2.51
Ph	6.5–8.5	–	7.5
Temperature (°C)	18–35 °C	12 °C	20 °C
CO_2_ (mg L^−1^)	0.70 ± 0.10	–	200
Total alkalinity (mg L^−1^)	25–500 ppm	–	680
Chloride (mg L^−1^)	250	–	350
Total hardness (mg L^−1^)	100–800 mg/L	–	420
Ca (mg L^−1^)	10–160 ppm	–	213.33
Total dissolved solids (mg L^−1^)	100–12,000	500	356.87
Total salinity	0–5	5	5.33 ± 1
Nitrate	100	20–50	67
Sodium	20	–	35
Phosphates	5	0–20	12
Fe	0.3	–	–
Pb	0.2	–	–
Ni	0.1	–	–
Co	0.03	–	–
Cu	1.0	–	–
Zn	1.0	–	–
As	0.05	–	–
Mn	1.0	–	–
Cr	0.1	–	–
Mg	1.0	–	–
Cd	0.1		

#### Heavy metal analysis

The results showed high mean values of all the metals in the industrial effluents collected from all the sub-sampling sites. In the present study, the higher concentrations of the Fe were analyzed in the effluents with a mean value of 90.52 ± 32.08 mgL^−1^. During present investigation, enormous loads of chromium were recorded from tannery effluents of all the sampling sites. The overall mean values of chromium were calculated for all the four sub-sampling sites (Textile industry, tannery industry, chemical industry-I & II) and were found to be 17.41 ± 0.1 mgL^−1^, 53.12 ± 1.00 mgL^−1^, 39.95 ± 1.50 mgL^−1^ and 12.30 ± 0.1 mgL^−1^, respectively. The chromium levels were statistically significantly higher in the tannery effluents as compared to the effluents of textile and chemical industries at *p* < 0.05 for the industrial influents collected from the four sub-sampling cities.

The mean values of Pb, Cr, Ni, As, Mn and Zn analyzed in the industrial effluents of SIE were 21.28 ± 6.85 mgL^−1^, 65.81 ± 40.01 mgL^−1^, 0.74 ± 0.80 mgL^−1^, 2.57 ± 2.60 mgL^−1^, 147.36 ± 80.91 mgL^−1^ and 62.90 ± 8.63 mgL-1, respectively. The analysis of textile effluents of SIE showed the order of concentration as follows: Mn > Fe > K > Zn > Na > Cr > Cd > Pb > Hg > As > Co > Cu > Ni whereas the same for tannery effluents of SIE was: Cr > Fe > K > Zn > Mn > Na > Cd > Hg > Pb > Co > As > Ni > Cu (Table 4).

**Table 4:** Chemical analysis of heavy metal from the Sunder industrial effluents.

Heavy metal levels (mg L^−1^)	Sampling sites				
	Textile industry (S1)	Tannery industry (S2)	Chemical industry-I (S3)	Chemical industry-II (S4)	Overall mean ± S.D
Fe	70 .54 ± 0.03^**a**^	45.64 ± 0.06^**c**^	52.99 ± 0.08^**b**^	26.32 ± 0.03^**d**^	90.52 ± 32.08
Cr	17.41 ± 0.1^**c**^	53.12 ± 1.0^**a**^	39.95 ± 1.50^**b**^	12.30 ± 0.1^**d**^	65.81 ± 40.01
Pb	10.07 ± 1.00^**bc**^	8.05 ± 0.02^**d**^	15.02 ± 1.0^**a**^	9.09 ± 0.01^**cd**^	21.28 ± 6.85
Cd	10.49 ± 1.0^**cd**^	12.23 ± 1.00^**b**^	17.0 ± 0.05^**a**^	10.25 ± 1.0^**d**^	25.65 ± 6.60
Ni	0.86 ± 0.01^**a**^	0.14 ± 0.01^**c**^	0.03 ± 0.03^**d**^	0.36 ± 0.02^**b**^	0.74 ± 0.80
Hg	10.05 ± 0.03^**ab**^	9.23 ± 0.07^**b**^	4.56 ± 0.01^**cd**^	3.28 ± 0.02^**d**^	6.78 ± 3.36
Cu	1.83 ± 1.0^**a**^	0.02 ± 0.01^**d**^	0.79 ± 0.01^**c**^	0.89 ± 0.01^**bc**^	1.45 ± 1.22
As	3.61 ± 0.10^**ab**^	2.12 ± 1.0^**b**^	0.16 ± 0.04^**d**^	0.57 ± 0.02^**c**^	2.57 ± 2.60
Zn	31.62 ± 1.0^**c**^	32.97 ± 10^**bc**^	26.09 ± 1.0^**d**^	35.21 ± 10^**ab**^	62.90 ± 8.63
Na	25.42 ± 0.10^**a**^	14.41 ± 1.0^**cd**^	12.37 ± 0.01^**d**^	22.25 ± 1.0^**b**^	34.96 ± 11.44
Mn	110.06 ± 0.01^**b**^	32.34 ± 2.26^**d**^	59.12 ± 1.0^**c**^	119.13 ± 1.0^**a**^	147.36 ± 80.91
K	57.25 ± 2.0^**a**^	42.78 ± 1.50^**b**^	33.06 ± 1.2^**c**^	28.41 ± 1.0^**d**^	83.19 ± 27.16
Co	3.55 ± 0.07^**ab**^	3.17 ± 0.03^**b**^	2.89 ± 1.0^**c**^	1.12 ± 0.10^**d**^	5.89 ± 1.41

Means with different letters in a single row are statistically non-significant at *p* < 0.05.

### Hematological and biochemical analysis

In the present study, blood biochemical and hematological parameters were measured up to 15 days after starting the exposure of sub lethal dose of LC_50_ of industrial effluents. The mean concentrations of Hb, RBCs, WBCs, derived erythrocyte indices (MCV and MCHC) and other parameters of *O. niloticus* are presented in Table 5.

**Table 5:** Hematological analysis of fish exposed to different concentrations of LC_50_ of Industrial Effluents of Sunder Industrial Estate, Lahore.

Hematological parameters	Control	Treated
RBCs × 10^6^/ul	0.44 ± 0.08	0.34 ± 0.15
WBCs × 10^3^/ul	2.53 ± 0.25	5.46 ± 0.25
Hb g/dl	3.3 ± 0.2	2.04 ± 0.03
Hct %	6.56 ± 1.17	2.43 ± 1.17
MCV Fl	70.22 ± 8.27	66.16 ± 5.19
MCHC g/Dl	104 ± 14.42	57.33 ± 4.04
Neutrophils %	19 ± 6	70 ± 9.16
Lymphocytes %	6.34 ± 5.68	66.00 ± 6.55
Monocytes %	4.50 ± 1	20.33 ± 4.51
Eosinophils %	3.00 ± 1	26.33 ± 6.02

Values are expressed as mean + S.D

All the samples were analyzed in triplicates.

The sub-lethal industrial effluents exposure to tilapia significantly reduced the levels of red blood cells (RBC), hematocrit (HCT), and hemoglobin (Hb) concentration, mean corpuscular volume (MCV) but increased the circulated levels of white blood cells (WBC) and lymphocytes compared to the negative control. The alterations observed in hematological parameters were observed in treated group as compared to the control group. Hb and RBC activities reduced as 0.62 fold and 0.77 fold, respectively in treated group, while derived erythrocyte indices (MCV and MCHC) were also reduced 0.94 fold and 0.55 fold in exposed group when compared to control. The irregularities in the WBCs profile for neutrophils, lymphocytes, monocytes and eosinophils were also prominent in the treated group exposed to 1/5th sublethal concentrations of industrial effluents when compared to the control group. A significant (*p* < 0.05) increase in WBCs was observed in treated specimens with 2.16 fold that revealed a potentially disturbed immune system of fish exposed to the sub-lethal (1/5th of LC_50_) industrial effluents of SIE. After exposure to the industrial effluents, lymphocytes showed highest significant (*p* < 0.001) rise in treatment group which is tenfold increase as compared to control group.

**Figure 2:**
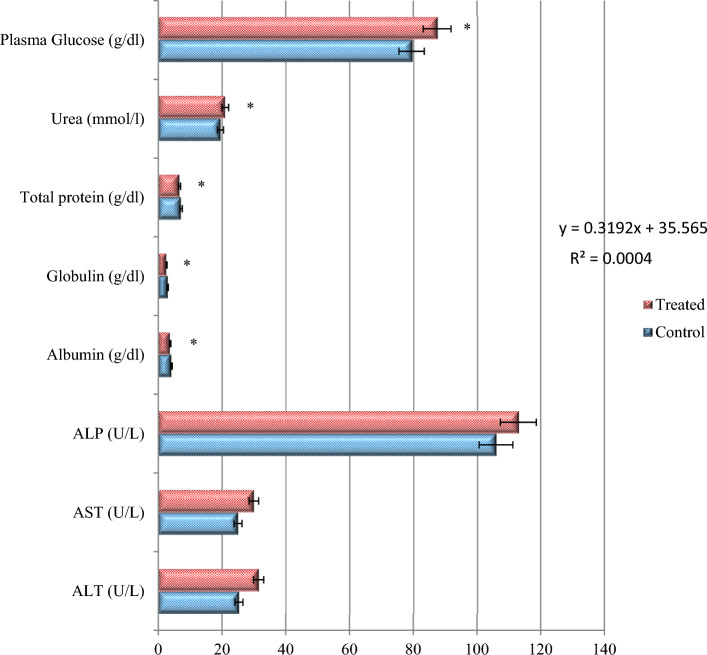
Effect of sublethal (1/5th LC_50_) of industrial effluents exposure on the biochemical parameters of *Oreochromis niloticus*. *Stands for significant differences among treatments compared with the control using two-way analysis of variance (ANOVA) followed by Tukey’s multiple comparisons (*p* < 0.05).

Regarding biochemical analysis, our finding showed that AST, ALT and ALP 20%, 25% and 7% respectively, were increased in treated groups due to the toxic effect of industrial effluents and to cope with the high energy demand to reduce the toxicity on fish body as compared to control group. Total protein content level recorded for the treated fish exposed to sub-lethal industrial effluents was -7% lower than control group. The albumin and globulin levels decreased -10% and -13% for the treatment group during the current investigation. Moreover, the concentrations of urea 8% and plasma glucose 10% were increased in treated group as compared to control group as shown in Fig. 2. AST, ALT and ALP are insignificant at *p* > 0.05, while remaining all parameters were statistically significant at *p* < 0.05 with F = 2.64.

### Effect of industrial effluents exposure on antioxidant defense system

**Figure 3:**
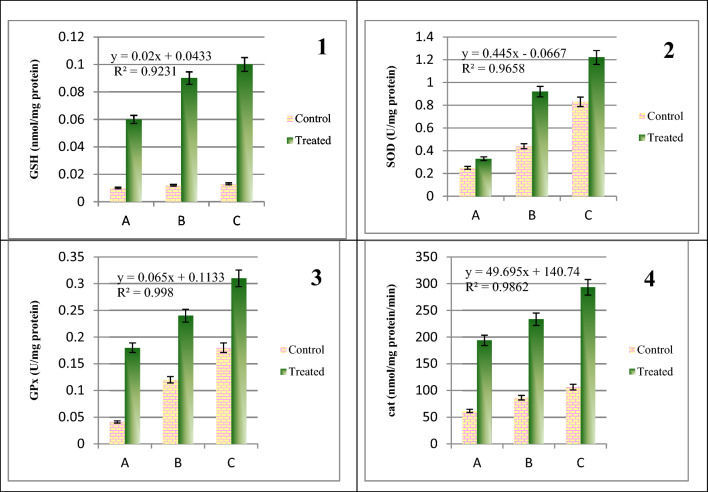
Level of antioxidant enzymes in brain homogenate of *O. niloticus* exposed to 1/5th of LC50 of Industrial Effluents of Sunder Industrial Estate, Lahore, (**1**) = Level of reduced glutathione (GSH), (**2**) = Level of reduced superoxide dismutase (SOD), (**3**) = Level of glutathione peroxidase (GPx) and (**4**) = Level of catalase (Cat), A, B and C are the replicates (n = 10).

Present study depicted that the activity of antioxidant enzymes (superoxide dismutase, catalase, and glutathione peroxidase) and reduced glutathione (non-enzymatic antioxidant) in the brain of fish exposed to sublethal concentration (1/5th LC_50_) of industrial effluents increased as compared to control. The increase of SOD (0.99 ± 0.24 U/mg protein) activity in brain was higher as 3.42 fold at the sub lethal concentrations of industrial effluents i.e. 1/5th of LC_50_ as compared to control. While other antioxidants enzymes CAT (206.79 ± 50.04 nmol/mg protein/min) and GPx (0.24 ± 0.06 U/mg protein) levels were also increased at different folds i.e. 2.44 and 4.8, respectively. But the highest activity of GSH (0.08 ± 0.02 nmol/mg protein/min) was observed in brain as eightfold increase in treated group as compared to the lower level of control group (Fig. 3). So SOD, CAT, GPx and GSH showed statistically significantly (*p* <  0.05) high values in treated group as compared to the non-treated control group.

### Histological analysis

**Figure 4:**
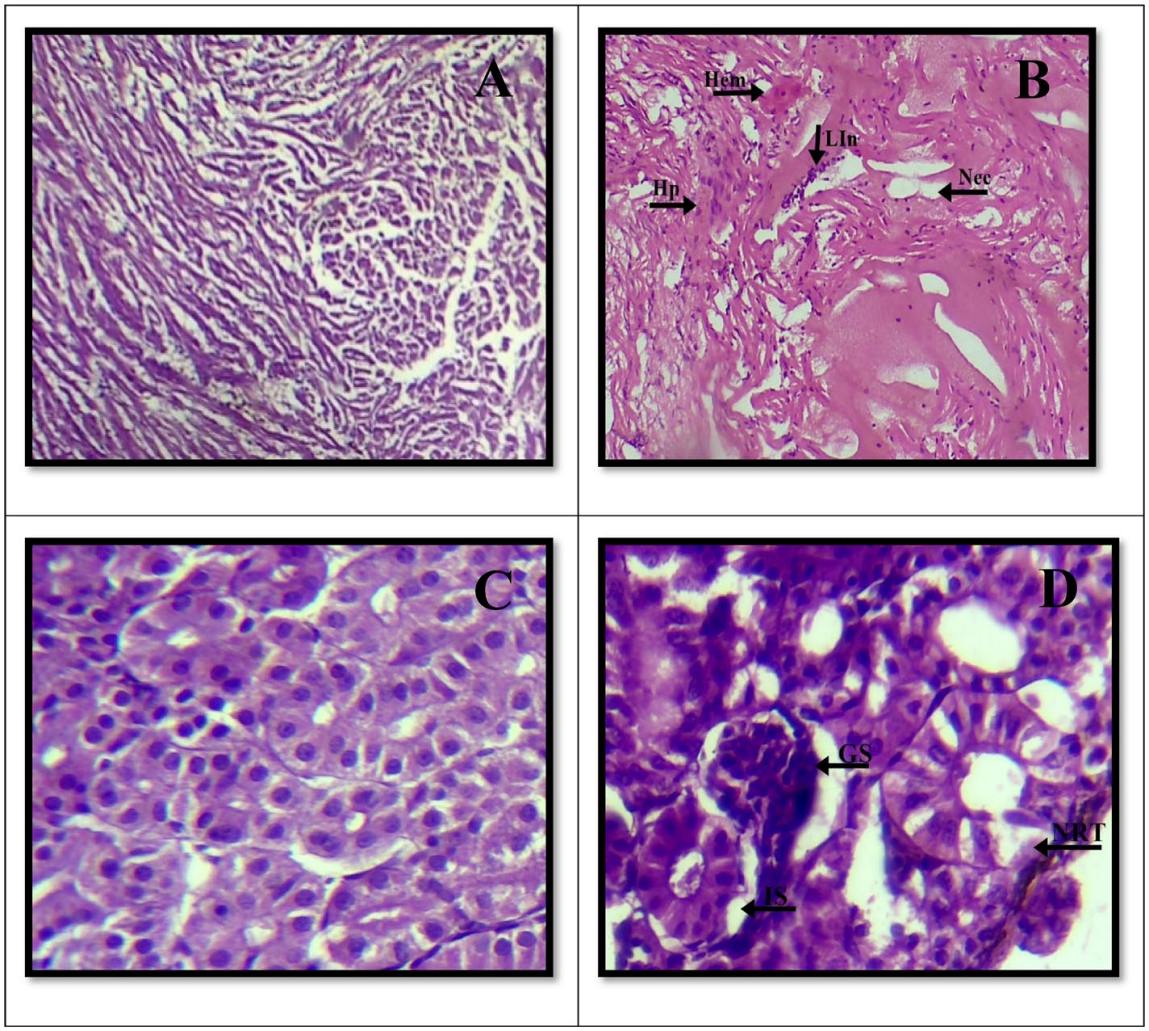
Histopathological alteration of transversely paraffin sectioned of Heart and Kidney of *Oreochromis niloticus* electron micrographs (10 × &40 ×) (H&E). (**A** & **C**) Control heart, kidney. (**B** &** D**) = exposed heart and kidney. Normal structure of kidney tissues was observed in the control fishes. Abbriviations are as = Hem = Hemorrhage, Lin = Leucocytes Infiltration, Nec = Necrosis, HP = Hyperplasia, GS = Glomerulus shrinkage, NRT = Necrotic renal tubule and IS = Increase Glomerulus space.

The kidney and heart tissues from the control group of fish were examined histopathologically, and they revealed typical histological features. The fish's heart and kidney tissues appeared to be in good health and to be operating well. However, many microscopic abnormalities and pathological alterations were visible when the heart and kidney tissues of fish exposed to a composite of sub-lethal industrial effluents. The histological changes in the cardiac tissues ranged from minor to sever alterations like hemorrhage, leucocyte infiltration, necrosis, lifting and bending of the pericardium and myocarditis (inflammation of the heart muscle). The exposed fish's kidney tissues also showed pathological alterations. These changes, which ranged in severity from mild to moderate, included a number of anomalies. These included necrotic renal tubules, shrinkage of glomeruli, hydropic swelling (abnormal swelling of kidney cells brought on by fluid accumulation), necrotic proximal tubules, and an increase in space within the glomeruli (Fig. 4).

## Discussion

The composition of industrial effluents varies from country to country depending on the type of industries, treatment strategies, manufacturing processes, equipment and chemicals used and the type of commodities produced^28^. It was reported^29^ that fish are appropriate experimental models for the evaluation of biomarkers of oxidative stress created by pollutants because they play a dual role as being on top of the aquatic food chain as vertebrates and respond strongly to stress conditions. The fish chosen for the current study was the freshwater fish, *O. niloticus*, an edible fish of Pakistan. Additionally, fish has gained a lot of significance because it has been discovered that exposure to industrial effluents causes major changes in haematological and histopathological indices, rendering fish unhealthy^30^.

Freshwater is impacted by a variety of toxins that result from untreated materials releases from factories, agriculture activities, and some everyday activities. This water pollution negatively affects aquatic life as well as humans^31^. Several vertebrates, even fish, have evolved methods and control mechanisms that enable them to absorb and use certain minerals. Untreated effluent's physicochemical parameters such as elevated pH, turbidity, ammonia, and electrical conductivity cause stress conditions that harm fish's gross structures. Different physicochemical characteristics from various industries, including pH, electrical conductivity, ammonia, etc., were also examined during the current investigation because they were out of range and stressing the fish. Total dissolved solid (TDS) was also high in different effluents collecting from different industries as compared to the control, result is parallel to^32^ studied physicochemical parameters in polluted canal.

However, metal determination of these effluents showed different concentration of metals in effluents. The results of current investigation in line to the findings of^33^ who analyzed the seven effluent samples collected from Savar industrial area in Bangladesh for heavy metals levels. The average concentration of Fe, Mn, Cr, Zn, As, Ni and Pb were 606.64, 72.71, 5.04, 25.05, 1.72, 2.37 and 1.56 mgL^−1^ in effluents, respectively. The order of average heavy metal content was Fe > Mn > Zn > Cr > Ni > As > Pb in effluent water. From the current study heavy metals concentration of textile effluents was given as: Mn > Fe > K > Zn > Na > Cr > Cd > Pb > Hg > As > Co > Cu > Ni. Heavy metal analysis from water of Yamuna River, India was also conducted by^34^. Heavy metals concentration from river water was given as: Fe > Mn > Zn > Cu > Ni > Cr > Cd.

Due to the toxicants present in industrial effluents cause oxidative stress in aquatic species. These toxins alter the physiology of fish, interfere enzyme activity, change the structure of cell organelles, and increase dangerous compound concentrations^35^. According to the study, stressed fish exposed to industrial effluents had more antioxidant enzyme activity than the control group, which was probably caused by the control group's lower ROS production. It is well established that exposure to industrial effluents increases ROS levels and thus boosts antioxidant activity. This outcome is consistent with observations made in *O. niloticus* collected from industrial polluted water of Rosetta Branch^36^. From current study, level of antioxidant percentage was given as: GSH > GPx > SOD > CAT, but dissimilar result obtained by^37^ in the case of GSH but similar in SOD and CAT in *Channa punctatus* collected from Panethi, polluted reservoir, India.

According to the study's findings, the liver's alanine and aspartate transaminase activity is elevated when exposed to untreated effluent's harmful effects compared to control medium. When L-amino acids are transferred for the gluconeogenesis of carbohydrates during anaerobic respiration to meet the energy demands under extended toxic stress from pollutants, transaminases (ALT and AST) play a significant role^38^. The current study's findings demonstrated a significant rise in transaminase activity in functionally distinct treatment and exposure group. The involvement of amino acids in the Kreb’s cycle cycle for ensuring the energy crisis during stress conditions may be the cause of the higher level of transaminases found in the liver of aquatic species like fish^39^. Results are consistent to^40^ which revealed higher level of AST and ALT in *O. niloticus* collected from polluted water of Yamuna river and in *Cirrhinus cirrhosis* ^41^ treated with different level of petroleum effluents and *Danio rerio*^42^ exposed at different concentration of alcohol industry.

ALT and AST are non-functional serum enzymes that are typically found in the liver and other tissues. Their presence in blood serum may provide information on organ failure or tissue damage^43^. Consequently, alterations in the activity of ALP may obstruct the basic metabolism inside the cell, which might result in development and progression in cell. The level of ALP is considered as a prominent tool for evaluating stress induced by contaminants such as industrial effluents in fishes^44^. Likewise, in present study remarkable elevation in the kinetics of this enzyme has been recorded when fish are exposed to untreated effluent. Parallel observation for elevation in ALP in *Heteropneustes fossilis*^25^ exposed to fertilizer industrial effluents. Urea and plasma glucose level of serum were increased in current study. The elevated in plasma glucose level reflects the enhanced glycogenolysis and showed that the fish is under stress and actively consuming its energy reserves, such as glycogen in the liver and muscles^45^. While increase in urea level due to the damage of renal tubules^46^. The results are also consistent with^47^ *Clarias gariepinus* collected from the contaminated area.

Moreover, total protein, albumin and globulin levels decreased in exposed fish as compared to control group. Significant decrease in total protein content indicates that, stress due to effluent treatment induces proteolysis to meet the increased demand^48^. Same finding for protein content was determined by^49^ who exposed *Labeo rohita* to tannery effluents. During hemorrhage or external injuries, there is a loss of protein, including albumin and globulin. The decline in total blood protein, albumin and albumin/globulin ratio, especially at sublethal dose of industrial effluents, is a reflection of the effluent's immunosuppressive and hepatic dysfunctional effects^46^. Parallel results was obtained at different interval of sampling in *Labeo rohita* exposed to thermal power station effluents^50^.

In the current study, it was shown that treated groups had lower levels of RBC, Hb, Hct, MCV and MCHC, but WBC, neutrophils, lymphocytes, monocytes, and eosinophils had sharply increased. Toxic exposure of industrial effluents may change the RBC count due to cells that are no longer viable, which then causes hemolysis and anemia^51^. There have been reports of decreased RBC, increased WBC and fluctuation in their indices counts in numerous fish species, i.e. *Clarias gariepinus* treated with pharmaceutical industrial effluents^52^ and *Cyprinus carpio* exposed to sewage effluent^53^. As we also observed a drop in Hb content, the current study's fall in MCHC points to either RBC swell or a reduction in hemoglobin production. The observed changes could be the result of the effluent elements' cumulative effects on the metabolic and hematological processes, which weaken the immune system. In fish exposed to toxic effluents, elevated leucocyte and lymphocyte counts show an enhanced immune response, showing elevated production of antibody for survival and recovery from contamination difficulties.^54^.

Industrial effluents cause minor to major kidney and cardiac histopathological alterations in test organisms. When compared to control fish, the heart of treated fish under exposure displayed significant alterations like hemorrhage, atrophy, pericardium injury, myocarditis, and necrosis, indicating that cardiac tissue is not resistant to this exposure. Consistent results with the current’s study was found in *Heteropneustes fossilis*^55^, depicting significant alterations in heart tissue after exposure to industrial effluents. While exposed kidney showed necrotic proximal tubules, damaged renal/glomerular tubules and dilated bowman capsule. The identified degree of tissue alteration values is consistent with previous studies that treated *Labeo rohita* with chronic wastewater exposure, as reported by^56^. Histopathological biomarkers are indicators of the population's general health in a biological system^57^.

## Conclusion

In current study Sunder Industrial Estate's industrial effluents were examined, and it was discovered that these wastewater sources had a significant potential for toxicity, surpassing the standard discharge limits. Histopathological, *B*iochemical and antioxidant enzyme profiles of *O. niloticus* showed cellular and organ damage, proving their susceptibility to industrial effluent. Adverse effects of such effluents disturb human health as well. As a result of this work, *Oreochromis niloticus* should be used in water quality monitoring programs.

## Data Availability

The data is available from the corresponding author, Ghazala Jabeen upon reasonable request.

## References

[1] Pappa J, Lenin T, Sundaram A, Kumar S (2016). Biochemical changes in estuarine fish *Mugil cephalus* exposed to industrial effluent. Int. J. Adv. Multi. Res..

[2] Mostafaie A, Cardoso DN, Kamali M, Loureiro S (2021). A scientometric study on industrial effluent and sludge toxicity. Toxics.

[3] Kaur N, Brraich OS (2022). Impact of industrial effluents on physico-chemical parameters of water and fatty acid profile of fish, Labeo rohita (Hamilton), collected from the Ramsar sites of Punjab, India. Environ. Sci. Pollut. Res..

[4] Chen C, Qian Y, Chen Q, Li C (2011). Assessment of daily intake of toxic elements due to consumption of vegetables, fruits, meat, and seafood by inhabitants of Xiamen, China. J. Food Sci..

[5] Javed M, Ahmad I, Usmani N, Ahmad M (2016). Bioaccumulation, oxidative stress and genotoxicity in fish (*Channa punctatus*) exposed to a thermal power plant effluent. Ecotoxicol. Environ. Saf..

[6] Ahmad I, Ahmad M (2016). Fresh water fish, Channa punctatus, as a model for pendimethalin genotoxicity testing: A new approach toward aquatic environmental contaminants. Environ. Toxicol..

[7] Souza FA, Dziedzic M, Cubas SA, Maranho LT (2013). Restoration of polluted waters by phytoremediation using Myriophyllum aquaticum (Vell.) Verdc., Haloragaceae. J. Environ. Manag..

[8] Patil A, Reddy P (2017). Endosulfan induced oxidative stress in Tilapia mossambica. Life Sci. Int. Res. J.

[9] Al-Asgah NA, Abdel-Warith A-WA, Younis E-SM, Allam HY (2015). Haematological and biochemical parameters and tissue accumulations of cadmium in Oreochromis niloticus exposed to various concentrations of cadmium chloride. Saudi J. Biol. Sci..

[10] Essien, E. B., & Chinwe, B. W. A. N. Assessment of the toxic effect of mixed effluents from trans-amadi industrial layout on Tilapia (Oreochromis niloticus) in Okrika River, Port Harcourt, Rivers State, Nigeria. *Assesment* **5** (2015).

[11] Gül Ş, Belge-Kurutaş E, Yıldız E, Şahan A, Doran F (2004). Pollution correlated modifications of liver antioxidant systems and histopathology of fish (Cyprinidae) living in Seyhan Dam Lake, Turkey. Environ. Int..

[12] Chen L (2021). Reactive oxygen species (ROS)-mediated regulation of muscle texture in grass carp fed with dietary oxidants. Aquaculture.

[13] Jerome FC, Hassan A, Omoniyi-Esan GO, Odujoko OO, Chukwuka AV (2017). Metal uptake, oxidative stress and histopathological alterations in gills and hepatopancreas of *Callinectes amnicola* exposed to industrial effluent. Ecotoxicol. Environ. Safety.

[14] Wang C (2021). Effect of Chronic exposure to textile wastewater treatment plant effluents on growth performance, oxidative stress, and intestinal microbiota in adult zebrafish (*Danio rerio*). Front. Microbiol..

[15] Samanta P (2018). Comparative assessment of the adverse outcome of wastewater effluents by integrating oxidative stress and histopathological alterations in endemic fish. J. Hazard. Mater..

[16] Chinnadurai K (2022). Toxicity evaluation and oxidative stress response of fumaronitrile, a persistent organic pollutant (POP) of industrial waste water on tilapia fish (*Oreochromis mossambicus*). Environ. Res..

[17] Abiona, O., Awojide, O., Odesanmi, S. & Ajayi, O. Assessment of heavy and trace metal contents of internal organs of tilapia (*Oreochromis niloticus*) and catfish (*Clarias gariepinus*) obtained from Dandaru fish pond, Ibadan Oyo State. **10** (2018).

[18] Fagbohun AF, Ola-Davies OE, Emikpe BO, Obagbemiro O, Adeyemo OK (2020). Chronic exposure to no-effect concentration of diazinon induced histological lesions in organs of *Clarias gariepinus*. Agric. Sci..

[19] EPA, U. Guidelines for ensuring and maximizing the quality, objectivity, utility, and integrity of information disseminated by the environmental protection agency. (2002).

[20] EPA, U. Understanding and accounting for method variability in whole effluent toxicity applications under the national pollutant discharge elimination system program. (EPA 833-R-00-003. Washington, DC: US Environmental Protection Agency, Office, 2000).

[21] Baird, R. B. Water environment federation, American public health association, American, (2017). 10.2190/NS.20.3.l 20943481

[22] Jabeen G (2018). Evaluation of fish health status and histopathology in gills and liver due to metal contaminated sediments exposure. Bull. Environ. Contam. Toxicol..

[23] Reish DL, Oshida PS (1987). Manual of methods in aquatic environment research.

[24] Javahery S, Nekoubin H, Moradlu AH (2012). Effect of anaesthesia with clove oil in fish (review). Fish Physiol. Biochem..

[25] Singh U, Pandey RS (2021). Fertilizer industry effluent induced hematological, histopathological and biochemical alterations in a stinging catfish, *Heteropneustes fossilis* (Bloch, 1794). Environ. Sustain. Indic..

[26] Busher JT (1990). Serum albumin and globulin. Clin. Methods: History Phys. Lab. Exam..

[27] Bernet D, Schmidt H, Meier W, Burkhardt-Holm P, Wahli T (1999). Histopathology in fish: Proposal for a protocol to assess aquatic pollution. J. Fish Dis..

[28] Ghaly A, Ananthashankar R, Alhattab M, Ramakrishnan VV (2014). Production, characterization and treatment of textile effluents: A critical review. J. Chem. Eng. Process. Technol..

[29] Doherty V, Ogunkuade O, Kanife U (2010). Biomarkers of oxidative stress and heavy metal levels as indicators of environmental pollution in some selected fishes in Lagos, Nigeria. Am. Eurasian J. Agric. Environ. Sci.

[30] Kumar Maurya P, Malik DS, Kumar Yadav K, Gupta N, Kumar S (2019). Haematological and histological changes in fish *Heteropneustes fossilis* exposed to pesticides from industrial waste water. Hum. Ecol. Risk Assess. Int. J..

[31] Elarabany N, Bahnasawy M (2019). Comparative and interactive biochemical effects of sub-lethal concentrations of cadmium and lead on some tissues of the African Catfish (*Clarias gariepinus*). Toxicol. Res..

[32] Javed M, Ahmad I, Usmani N, Ahmad M (2016). Studies on biomarkers of oxidative stress and associated genotoxicity and histopathology in Channa punctatus from heavy metal polluted canal. Chemosphere.

[33] Faisal B, Majumder RK, Uddin MJ, Halim M (2014). Studies on heavy metals in industrial effluent, river and groundwater of Savar industrial area, Bangladesh by principal component analysis. Int. J. Geomat. Geosci..

[34] Mahamood M (2021). Labeo rohita, a bioindicator for water quality and associated biomarkers of heavy metal toxicity. npj Clean Water.

[35] Vinodhini R, Narayanan M (2009). Biochemical changes of antioxidant enzymes in common carp (*Cyprinus carpio* L.) after heavy metal exposure. Turkish J. Vet. Anim. Sci..

[36] Khalil MT, Gad NS, Ahmed NAM, Mostafa SSE-D (2017). Antioxidant defense system alternations in fish as a bio-indicator of environmental pollution. Egyptian J. Aquatic Biol. Fish..

[37] Javed M, Ahmad MI, Usmani N, Ahmad M (2017). Multiple biomarker responses (serum biochemistry, oxidative stress, genotoxicity and histopathology) in *Channa punctatus* exposed to heavy metal loaded waste water. Sci. Rep..

[38] Tamizhazhagan V, Pugazhendy K (2016). The toxicity effect of Monocrotophos 36% EC on the Biochemical changes *Labeo rohita* (Hamilton, 1882). Int. J. Sci. Res. Dev..

[39] Tiwari RK, Singh S, Pandey RS (2019). Assessment of acute toxicity and biochemical responses to chlorpyrifos, cypermethrin and their combination exposed earthworm, *Eudrilus eugeniae*. Toxicol. Rep..

[40] Khan MS, Javed M, Rehman MT, Urooj M, Ahmad MI (2020). Heavy metal pollution and risk assessment by the battery of toxicity tests. Sci. Rep..

[41] Hamidi S (2022). Effect of petroleum wastewater treated with gravity separation and magnetite nanoparticles adsorption methods on the blood biochemical response of mrigal fish (*Cirrhinus cirrhosus*). Environ. Sci. Pollut. Res..

[42] Derikvandy A (2020). Genotoxicity and oxidative damage in zebrafish (*Danio rerio*) after exposure to effluent from ethyl alcohol industry. Chemosphere.

[43] Essien, E. B. & Chinwe, B. Assessment of the toxic effect of mixed effluents from trans-amadi industrial layout on Tilapia (*Oreochromis niloticus*) in Okrika River, Port Harcourt, Rivers State, Nigeria. *Assessment* **5** (2015).

[44] Al-Ghanim KA (2020). Sub-lethal effect of synthetic pyrethroid pesticide on metabolic enzymes and protein profile of non-target Zebra fish, *Danio rerio*. Saudi J. Biol. Sci..

[45] Fırat O (2011). A comparative study on the effects of a pesticide (cypermethrin) and two metals (copper, lead) to serum biochemistry of Nile tilapia, *Oreochromis niloticus*. Fish. Physiol. Biochem..

[46] Srivastava B, Reddy P (2020). Haematological and serum biomarker responses in *Heteropneustes fossilis* exposed to bisphenol A. Nat. Environ. Pollut. Technol..

[47] Zaghloul KH, Mohamed HA, Abdullatef AM, Khalil MW (2020). Genotoxic and Histopathological effects of water pollution on *Clarias gariepinus* fish at fayoum governorate. Egypt. Nat. Resour..

[48] Kumar R, Banerjee TK (2016). Arsenic induced hematological and biochemical responses in nutritionally important catfish *Clarias batrachus* (L.). Toxicol. Rep..

[49] Muley DV, Karanjkar DM, Maske SV (2007). Impact of industrial effluents on the biochemical composition of fresh water fish *Labeo rohita*. J. Environ. Biol..

[50] Anant D, Suresh Z (2015). Thermal power station effluent induced biochemical changes in the blood of freshwater fish, *Labeo rohita*. Int. J. Life Sci..

[51] Mul, J. D., Stanford, K. I., Hirshman, M. F. & Goodyear, L. J. in *Progress in Molecular Biology and Translational Science* Vol. 135,(ed Claude Bouchard) 17–37 (Academic Press, 2015). 10.1016/bs.pmbts.2015.07.020 PMC4727532 26477909

[52] Alimba CG, Adekoya KO, Soyinka OO (2019). Exposure to effluent from pharmaceutical industry induced cytogenotoxicity, hematological and histopathological alterations in *Clarias gariepinus* (Burchell, 1822). Excli. J..

[53] Banaee M, Tahery S, Vaziriyan M, Shahafve S, Nemadoost-Haghi B (2015). Reproductive health indicators of immature common carp exposed to municipal wastewater of Behbahan, Iran. J. Adv. Environ. Health Res..

[54] Schreck, C. B. & Tort, L. in *Fish Physiology* Vol. 35 (eds Carl B. Schreck, Lluis Tort, Anthony P. Farrell, & Colin J. Brauner) 1–34 (Academic Press, 2016).

[55] Maurya, P. K., Malik, D., Yadav, K. K., Gupta, N. & Kumar, S. Haematological and histological changes in fish Heteropneustes fossilis exposed to pesticides from industrial waste water. *Human and Ecological Risk Assessment: An Int. J.* (2019).

[56] Kaur R, Dua A (2016). Induction of histopathological lesions in renal tissue of the fish *Labeo rohita* upon exposure to municipal wastewater of Tung Dhab Drain, Amritsar, India. Turkish J. Zool..

[57] Khoshnood Z, Mokhlesi A, Khoshnood R (2010). Bioaccumulation of some heavy metals and histopathological alterations in liver of Euryglossa orientalis and Psettodes erumei along North Coast of the Persian Gulf. Afr. J. Biotechnol..

